# Assessment of Behavioral Characteristics With Procedures of Minimal Human Interference in the *mdx* Mouse Model for Duchenne Muscular Dystrophy

**DOI:** 10.3389/fnbeh.2020.629043

**Published:** 2021-01-20

**Authors:** Sarah Engelbeen, Annemieke Aartsma-Rus, Bastijn Koopmans, Maarten Loos, Maaike van Putten

**Affiliations:** ^1^Department of Human Genetics, Leiden University Medical Center, Leiden, Netherlands; ^2^Sylics (Synaptologics B.V.), Amsterdam, Netherlands

**Keywords:** cognitive abnormalities, dystrophin (DMD), behavior, cognitive flexibility, spatial working memory, anxiety

## Abstract

Duchenne muscular dystrophy (DMD) is a severe, progressive neuromuscular disorder caused by mutations in the *DMD* gene resulting in loss of functional dystrophin protein. The muscle dystrophin isoform is essential to protect muscles from contraction-induced damage. However, most dystrophin isoforms are expressed in the brain. In addition to progressive muscle weakness, many DMD patients therefore also exhibit intellectual and behavioral abnormalities. The most commonly used mouse model for DMD, the *mdx* mouse, lacks only the full-length dystrophin isoforms and has been extensively characterized for muscle pathology. In this study, we assessed behavioral effects of a lack of full-length dystrophins on spontaneous behavior, discrimination and reversal learning, anxiety, and short-term spatial memory and compared performance between male and female *mdx* mice. In contrast to our previous study using only female *mdx* mice, we could not reproduce the earlier observed reversal learning deficit. However, we did notice small differences in the number of visits made during the Y-maze and dark-light box. Results indicate that it is advisable to establish standard operating procedures specific to behavioral testing in *mdx* mice to allow the detection of the subtle phenotypic differences and to eliminate inter and intra laboratory variance.

## Introduction

Duchenne muscular dystrophy (DMD) is an X-linked neuromuscular disorder that is characterized by progressive muscle weakening and wasting. About 1 in 5,000 newborn boys are affected by this disease (Ricotti et al., 2016). DMD patients lack the protein dystrophin, which connects the cytoskeleton with the extracellular matrix, and thereby protects muscle fibers from damage during contractions. In the absence of dystrophin, chronic muscle damage and inflammation, and impaired regeneration lead to replacement of muscle tissue by connective and adipose tissue (Deconinck and Dan, 2007). Most DMD patients lose ambulation before their teens and die in their third to fourth decade of life due to respiratory and cardiac complications (Aartsma-Rus et al., 2006).

DMD is caused by frame-shift or nonsense mutations in the *DMD* gene, which is the largest known gene in the human genome. It contains 2.2 Mb of genomic DNA and 79 exons that make up the coding sequence for full-length isoforms. There are seven promoters spread over the *DMD* gene that give rise to several dystrophin isoforms (Aartsma-Rus et al., 2006). Full-length dystrophin isoforms (Dp427c, Dp427m, and Dp427p) are lost in all DMD patients and, depending on the position of the mutation, one or multiple shorter isoforms are lacking as well. In muscle, only the full-length isoform Dp427m is expressed, while the majority of the other isoforms (Dp427c and p, Dp140, Dp71 and Dp40) are primarily expressed in the brain (Aartsma-Rus et al., 2006; Doorenweerd et al., 2017).

It is therefore not surprising that the absence of dystrophin in the brain also has implications on brain development, behavior and intellect. Many DMD patients have learning difficulties and delayed developmental milestones. There is a higher incidence of comorbidities with neuropsychiatric and behavioral disorders such as autism-spectrum disorder, attention-deficit hyperactivity disorder, obsessive-compulsive disorder, anxiety, and depression (Hinton et al., 2006; Waite et al., 2012; Banihani et al., 2015; Ricotti et al., 2016). DMD patients often have an intellectual disability, and an IQ of one standard deviation below the mean of the general population (Hinton et al., 2006; Banihani et al., 2015).

The most commonly used DMD mouse model is the *mdx* mouse. This strain lacks the full-length dystrophin isoforms due to a spontaneous point mutation in exon 23 of the *Dmd* gene. In contrast to DMD patients, muscle pathology of the *mdx* mouse is not severe and they have a near normal life expectancy (Chamberlain et al., 2007). Yet, it has been demonstrated that also *mdx* mice show emotional defensive behavioral abnormalities and impaired long-term memory retention due to the absence of dystrophin (Vaillend et al., 2004; Vaillend and Chaussenot, 2017; Comim et al., 2019). An interesting finding is that *mdx* mice show a freeze response upon fixation or a stress stimuli (i.e., foot-shock), which is rarely seen in wild-type mice (Sekiguchi et al., 2009; Vaillend and Chaussenot, 2017; Comim et al., 2019).

A study by Remmelink et al. investigated performance in several cognitive domains in female *mdx* mice. In an automated home-cage (PhenoTyper) setup, a relatively strong and distinct impairment in reversal learning (RL) following a discrimination learning (DL) task was observed (Remmelink et al., 2016). The aim of this study was to test whether this particular phenotype can also be found in *mdx* males, or if dystrophin deficiency affects behavior differently in male and female mice. Interestingly, we could not reproduce these abnormalities in cognitive flexibility in neither *mdx* males nor females. Since the automated home-cage system avoids human-animal interaction and ensures a standardized housing and testing environment, we conclude that this irreproducibility must be attributable to sources of variation that acted prior to cognitive testing. Therefore, we investigated both studies in detail and report here a number of factors that may have contributed to irreproducibility. This finding highlights that behavioral tests are subject to variation, identified potential sources of variation, and supports the notion that behavioral studies ideally deliberately incorporate potential sources of variation in order to test reproducibility and hence the robustness of the findings.

## Materials and Methods

### Mice and Timeline of Behavioral Testing

Fifteen male and female *mdx* (C57BL/10ScSn-*mdx*/J) and fifteen male and female wild-type (C57BL/10ScSnJ) mice were bred from homozygous *mdx* and wild-type lines, respectively at the animal facility of the Leiden University Medical Center (Leiden, The Netherlands). Mice were housed in individually ventilated cages (Green line, Sealsafe Plus GM500, Tecniplast, Italy) with 12 h light/dark cycles, and had *ad libitum* access to water and standard RM3 chow (SDS, Essex, United Kingdom) under SPF (FELASA 2014) conditions. At the age-range of 8–16 weeks, mice were transported as one cohort to the behavioral facility of the VU University (Amsterdam, The Netherlands). Upon arrival, mice were kept in individually ventilated cages (Green line, Sealsafe Plus GM500) on sawdust bedding and enriched with cardboard nesting material in the same social groups and from each cage a blood sample of 1 mouse was taken to confirm health status. Four weeks later, after confirmation of the SPF (FELASA 2014) health status, mice were moved to a different floor where behavioral experiments were carried out. Here, mice were housed in Makrolon type II conventional open cages (Type 2 short, model 1284 or 1264, Tecniplast, Italy) on sawdust bedding and enriched with cardboard nesting material. Over the course of the experiments, males were housed individually, while females were socially housed in groups of 2–3 mice per cage, except for when mice went into the PhenoTyper cages in which all mice are individually housed. At the facility of VU University, mice were housed with 12 h light/dark cycles and were *ad libitum* provided with water and regular chow (2018 Teklad, Harlan Laboratories, Horst, the Netherlands). The experiments were approved by the Animal Ethics Committee of VU University and performed according to Dutch regulation for animal experimentation, and in accordance with EU Directive 2010/63/EU.

**Figure 1:**
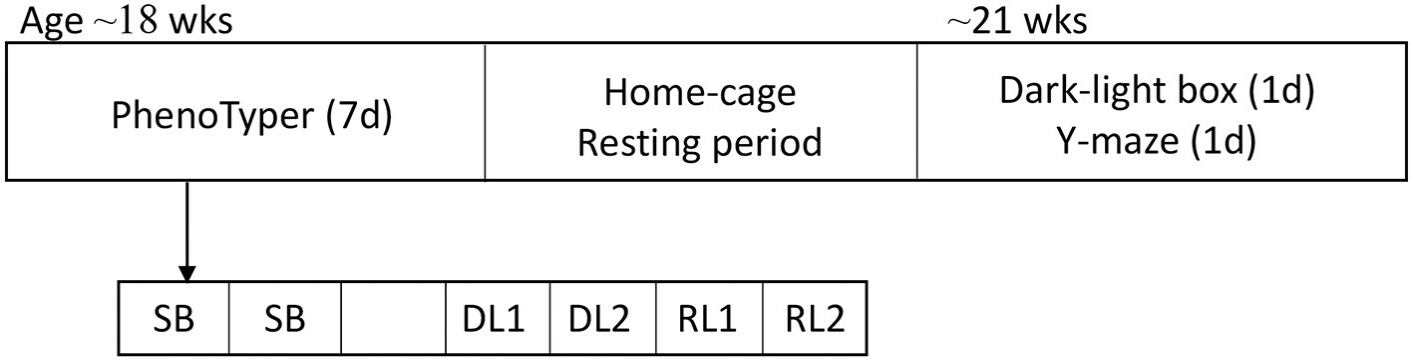
Overview of behavioral tests. Mice started at the age of ~18 weeks in the PhenoTyper home-cage in which spontaneous behavior was tracked for 2.5 days. After the spontaneous behavior assessment, mice were subjected to the DL/RL task for 4 days. After a resting period of 1–2 weeks, the DLB and the Y-maze tests were executed. SB, spontaneous behavior; DL, discrimination learning; RL, reversal learning; DLB, dark-light box.

To accommodate and standardize differences in age at behavioral testing, mice were assigned to 3 different batches, with the oldest mice in the first batch and youngest mice in the last batch. Mice were subjected to a series of experiments. First, at an average age of 18 weeks (youngest 16 weeks, oldest 21 weeks) mice were housed in PhenoTyper home-cages for seven days in which their spontaneous behavior was tracked with a camera-system for two and a half days, followed by a four-day discrimination and learning phase experiment to assess cognitive flexibility. Mice then had a resting period of 1–2 weeks. Thereafter, dark-light box and Y-maze tests were done on two consecutive days. Mice were sacrificed one to three days after the Y-maze test (Figure 1).

### PhenoTyper Home-Cage

The PhenoTyper (model 3000, Noldus Information technology, Wageningen, the Netherlands) is an observation home-cage in which mouse behavior can be automatically tracked by integrated cameras, and hardware actions can be initiated through various triggers using the video tracking software controlling the cages (EthoVision XT version 11, Noldus Information Technology, Wageningen, the Netherlands). Cages (Length (L) = 30 cm x Width (W) = 30 cm x Height (H) = 35 cm) are made from transparent Perspex walls with an opaque Perspex floor covered with bedding based on cellulose. The cages are equipped with a water bottle, a food grid and a triangular-shaped shelter, fixed in one corner, that has two entrances and is made from non-transparent material (W = 17.5 cm, H = 10 cm, diameter entrances = 9 cm). In the opposite corner, a reward pellet dispenser protruded with an aluminum tube into the cage. During the DL and RL tests an opaque Perspex wall (CognitionWall^TM^, Noldus Information Technology, Wageningen, the Netherlands) with three entrances was placed in front of the pellet dispenser (W = 17 cm, H = 25 cm, diameter entrances = 3.3 cm, Supplementary Figure 1).

### Spontaneous Behavior—Automated Home-Cage

Mice were housed in PhenoTyper cages during the light phase and video tracking started at 19:00 at the onset of the dark phase and continued for two and a half days to assess spontaneous behavior until 7:00 at the onset of the light phase of the third day in the cage. Regular chow and water were provided *ad libitum* during this period. Total distance moved per hour was extracted from the video tracking data. In addition, seventeen key parameters of spontaneous behavior (Loos et al., 2014) were extracted from the video tracking data. These parameters describe six domains; kinematic parameters of movement, shelter segments, habituation effects across days, the effect of the light/dark phase, anticipation of, and response to, light/dark transitions and activity bout characteristics (Supplementary Table 1).

### Discrimination and Reversal Learning—Automated Home-Cage

At 10:00 on the morning when the recording of spontaneous behavior has finished, regular chow was removed from the food grid and any remaining food on the cage floor was removed. At this moment, the pellet dispensers were connected to the cage and a few food rewards (Dustless Precision Rodent Pellets, 14 mg, F05684, Bio-Serv, Flemington, NJ, USA) were dispensed into the cage to let the mice habituate to the smell and taste of these food rewards. At 16:00–16:30, a wall with three entrances was placed in front of the pellet dispenser in the PhenoTyper cage and at 16:30, the DL and RL task protocol was started (Supplementary Figure 1). During the two-day DL task, mice had to learn to earn their food by entering the left entrance of the wall. Entries through the middle and right entrance were registered, but not rewarded. Entrance bias was assessed for the first 30 entries in the DL task, during which no rewards were dispensed. Entries are registered once the mouse body, defined by more than half of the body, moves away from the entrance to avoid multiple entry registrations when mice remain inside an entrance during the experiment. The RL task followed immediately after the DL task and commenced at 19:00 on the 3rd day of the DL/RL task, i.e. 50.5 h after the start of the DL protocol. The RL task used the same protocol, however, now the right instead of the left entrance was rewarded. During both tasks, a pellet was dispensed for every fifth entry through the correct entrance (FR5 schedule of reinforcement).

To assess learning during DL and RL, the number of entries to reach a criterion of 80% correct entries in a moving window of the last 30 entries was calculated and plotted in a survival plot. During RL, perseverative errors were assessed by counting the number of entries in the previous rewarded entrance. The entries through the middle entrance were used as a measure of neutral errors. Activity was assessed as the total number of entries and the total distance moved during DL and RL.

### Dark-Light Box Test

The dark-light box (DLB) procedure was performed as in our previous study (Remmelink et al., 2016). The DLB consists of a light and a dark compartment of equal sizes (L = 25 cm x W = 25 cm x H = 30 cm) separated by a motorized door. In this test, a mouse was introduced to the dark compartment (<10 Lux) and after 1 min the motorized door was opened and the mouse allowed to explore the light compartment (625 Lux). The motorized door was left open for 10 min and during this period the mouse's activity was tracked using the Viewer 2 software (BIOBSERVE GmbH, Bonn, Germany). The mouse was considered to be in the light compartment when its body center-point was at least 2 cm away from the door. Anxiety was assessed by the latency to light, the time spent in the light and the number of visits to the light compartment.

### Y-Maze Spontaneous Alteration Test

Short-term spatial memory and alternation was assessed in a Y-maze (white PVC, arms L = 40 cm x W = 9.5 cm, H = 15.5 cm). A single white fluorescent light bulb illuminated the Y-maze. Mice were placed in the center of the maze and behavior was tracked for 10 min (Viewer 2, BIOBSERVE GmbH, Bonn, Germany). Spontaneous alterations were automatically recorded utilizing the Viewer 2 tracking software. Hereto, the Y-maze was divided into a center zone and three arm zones. The border between the center and arm zones was set at 3 cm into the arms. Zone visits were counted when both the nose and center of gravity crossed the zone border. Spontaneous alternation percentage was defined by the number of times a mouse visited all three arms without revisiting one of the arms (correct triad) divided by the total number of possible triads, three consecutive arm visits in a clockwise or counterclockwise manner (i.e., ABC, ACB, BCA, etc.). Re-entries in the previously visited arm were included in the calculation of spontaneous alternation percentage resulting, resulting in a chance level of 22.2% (2/9; Holcomb et al., 1999). Indeed, permutation analyses of the arm visits of arbitrarily selected mice resulted in a spontaneous alternation percentage 25 ± 7%, nicely corresponding to theoretical chance level.

### Data Analysis

Data analyses were performed with GraphPad Prism (GraphPad Software, San Diego, California USA, version 8.1.1). Spontaneous behavior in the PhenoTyper cage was analyzed using the AHCODA data analysis platform (Sylics, Synaptologics B.V., Amsterdam, the Netherlands) after which the computed values were used for statistical evaluation.

All data was assessed for normality. Some spontaneous behavior parameters and the latency to visit the light and visits to the light compartment during DLB were not normally distributed for one or more groups. A non-parametric test was performed for these analyses.

Total distance moved and key parameters for spontaneous behavior were analyzed using a one-way analysis of variance (ANOVA) test to assess variance between groups; differences between *mdx* and wild-type mice for each gender were assessed with Bonferroni's multiple comparison test. In case of non-normality of the data, Kruskal-Wallis test was performed followed by a Dunn's multiple comparison test to compare between wild-type and *mdx* for each gender (Supplementary Table 1).

Entries needed to reach the 80% criterion in DL and RL tasks were analyzed with the Mantel-Cox test. A survival plot is used so that mice that do not reach the 80% criteria can also be included and visualized on the graph. The effect of genotype, gender and day on perseverative and neutral errors during RL were assessed with the three-way ANOVA. Total entries and total distance moved were analyzed with the one-way ANOVA; variances between *mdx* and wild-types were analyzed with the Bonferroni's multiple comparison test (Supplementary Table 2).

Latency and number of visits to the light compartment during the DLB were analyzed using the Kruskal-Wallis test. Differences between *mdx* and wild-type groups were compared with Dunn's multiple comparison test. Time spent in the light compartment was analyzed with a one-way ANOVA for all groups. Differences between *mdx* and wild-type groups were compared with Bonferroni's multiple comparison test (Supplementary Table 2).

Spontaneous alternation percentage in the Y-maze was analyzed using one-sample *t*-tests comparing the different groups to chance levels of 25%. Total arm entries were analyzed with a one-way ANOVA for variance between groups while variance between *mdx* and wild-type was tested with the Bonferroni's multiple comparison test (Supplementary Table 2).

All data are presented as mean ± standard deviation. A *P*-value of < 0.05 was considered significant.

## Results

Mice (*n* = 15 per gender per genotype) were tested in three batches starting with the PhenoTyper home-cages at an age of 18 ± 1.3 weeks and the DLB/Y-maze at an age of 21 ± 2.2 weeks. From the spontaneous behavior and DL/RL data, one *mdx* male was excluded by automatic quality control since the mouse was sleeping outside of the shelter and the data file of one *mdx* female lost. One wild-type female was found dead before the start of the experiments. One *mdx* male and one female were found dead before the start of the DLB and Y-maze test. While *mdx* mice are more sensitive to stress, it seems unlikely to be the cause of death since the *mdx* mice were found during the resting period and did not undergo extensive handling during this period.

### Normal Spontaneous Behavior in an Automated Home-Cage

Spontaneous behavior was assessed in an automated home-cage for a duration of 2.5 days. No difference was found in the total distance moved between wild-type and *mdx* males, while wild-type females traveled a significantly larger distance than *mdx* females (Supplementary Figure 2).

A set of seventeen key parameters of spontaneous behavior were also studied. These parameters describe kinematics of movement, shelter segments, habituation effects across days, the effect of the light/dark phase, anticipation of, and response to, light/dark transitions and activity bout characteristics; and can detect changes in spontaneous behavior due to genetic differences (Loos et al., 2014). Gender differences and genotype differences were found for multiple parameters of spontaneous behavior (Supplementary Table 1). In male mice, no differences were found for key spontaneous behavior parameters. However, female mice showed differences in parameters for sheltering behavior. Based on the frequency distribution of the duration of shelter visits, it is possible to distinguish short, intermediate and long shelter visits. The short shelter visit threshold defines the upper limit of what is considered a short visit and this threshold was significantly lower in the wild-type females than in the *mdx* females (*P* = 0.0002). The long shelter visit threshold defines the threshold between intermediate and long shelter visits and this was also significantly lower in the wild-type females compared to *mdx* females (*P* = 0.0141, Supplementary Table 1). This indicates that female wild-types visited their shelter more frequently for shorter periods of time than female *mdx* mice.

### Unaltered Discrimination and Reversal Learning in *mdx* Mice

Cognitive flexibility was assessed using a food-based reward task in the PhenoTyper home-cage. A CognitionWall with three entries was placed in front of the food pellet dispenser and standard food was removed. Mice had to learn that every fifth entry through the correct entrance led to a pellet reward, while passing through the other entrances did not. All mice showed a preference for the left entrance during the first 30 entries made of DL, except for female *mdx* mice (Supplementary Figure 3).

**Figure 2:**
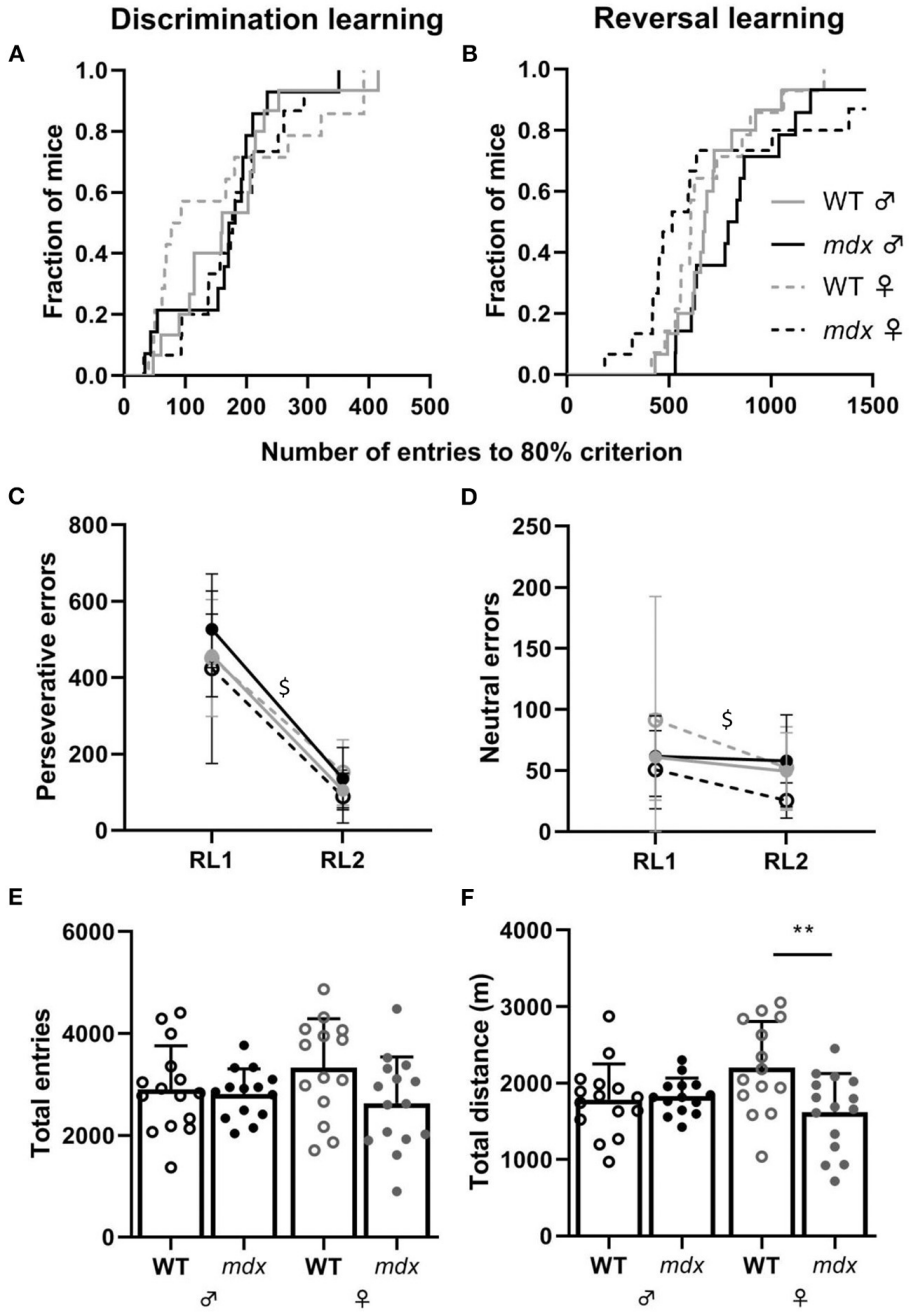
Performance in the DL and RL tasks. **(A)** Kaplan-Meier plot of DL showing the fraction of mice that reached the 80% performance criterium and the total number of entries needed to reach the criterium (WT ♂ *n* = 15; *mdx* ♂ *n* = 14; WT ♀ *n* = 14; *mdx* ♀ *n* = 15). **(B)** Kaplan-Meier plot of the proportion of mice reaching the 80% performance criterium for the RL task. One wild-type and *mdx* male and two *mdx* females did not reach the 80% criterion. **(C,D)** Number of perseverative and neutral errors made during day one (RL1) and day two (RL2) of the RL test. Errors were comparable for all groups for each day. A significant decline in perseverative (*P* ≤ 0.0001) and neutral (*P* = 0.0097) errors was observed during RL2 (visualized with $). **(E)** Total number of entries made through the CognitionWall during the entire DL and RL task. **(F)** Total distance moved in meters (m) during the entire DL and RL task. WT; wild-type. ** indicates *P* < 0.01.

During DL, all mice reached the 80% performance criterion. There was no difference between *mdx* and wild-type mice, nor genders, in the numbers of entries needed to reach this 80% criterion (Figure 2A). During the subsequent RL phase, the correct entrance was changed to the opposite entrance and mice, regardless of their genotype or gender, needed more entries to reach the 80% performance criterion (Figure 2B). One wild-type and *mdx* male and two *mdx* females did not reach the 80% criterion. While *mdx* and wild-type mice did not differ in the amount of perseverative errors they made during the first day of reversal learning (RL1), for all groups, there was a significant decrease in the amount of perseverative errors made when comparing the first and last day of RL (RL2) (*P* < 0.0001, Figure 2C). The amount of neutral errors mice made during the RL task also significantly dropped between RL1 and RL2 (*P* = 0.0097, Figure 2D).

We used total distance moved and total entries made as an assessment of the activity of mice during the DL/RL test. Total entries made is a more accurate measure of activity since the distance mice move behind the CognitionWall and inside the shelter are not measured. No differences were observed in the total amount of entries mice made during the DL and RL tests between the groups (Figure 2E). *Mdx* and wild-type males did not show a difference in the total distance moved during the DL/RL task whilst female wild-types moved significantly more than *mdx* females (*P* = 0.0095, Figure 2F). We did not assess differences between genders and genotypes for the light and dark phases during the DL/RL task. The idea of the PhenoTyper home-cage is that mice can perform the task whenever they want. Since mice are active during the night, most, if not all, entries will be made during the dark phase.

### No Anxiety-Related Behavior Found in *mdx* Mice With the Dark-Light Box

**Figure 3:**
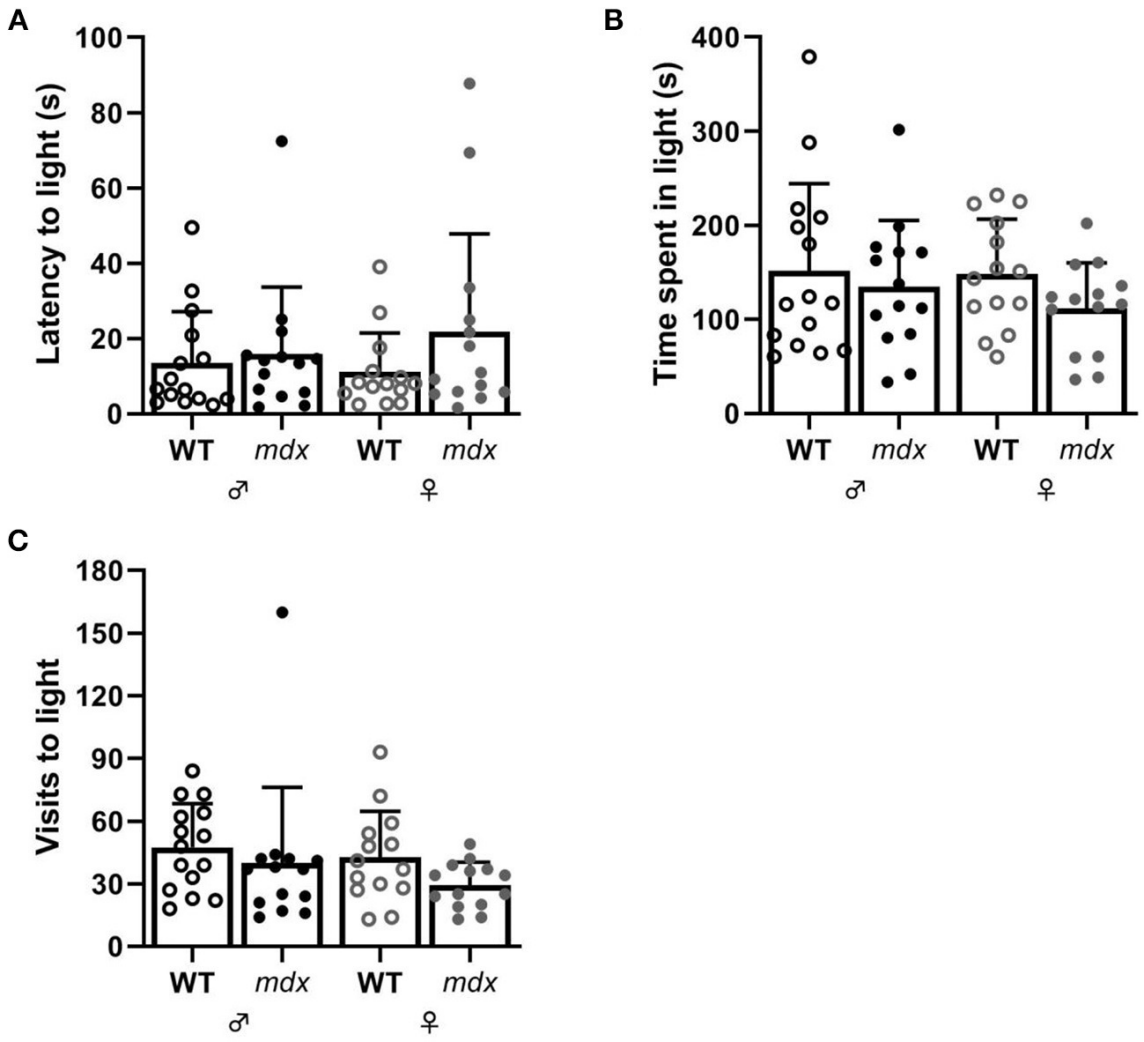
Measures of anxiety-related behavior in the DLB. **(A)** Latency to visit the light compartment in seconds. **(B)** Time spent in the light compartment. **(C)** Number of visits made to the light compartment. WT ♂ *n* = 15*; mdx* ♂ *n* = 14; WT ♀ *n* = 14; *mdx* ♀ *n* = 14.

Anxiety-related behavior was assessed in the DLB test. Mice were placed in a dark compartment, after 1 min a door opened to an equally sized brightly lit compartment and mice's explorative behavior was assessed in this light compartment as measure of anxiety. Wild-type and *mdx* mice did not show significant differences in the time it took before they entered the light compartment, the amount of time they spent in the light compartment and the number of visits to the light compartment (Figures 3A–C).

### Short-Term Spatial Memory Is Not Affected in *mdx* Mice in The Y-Maze

**Figure 4:**
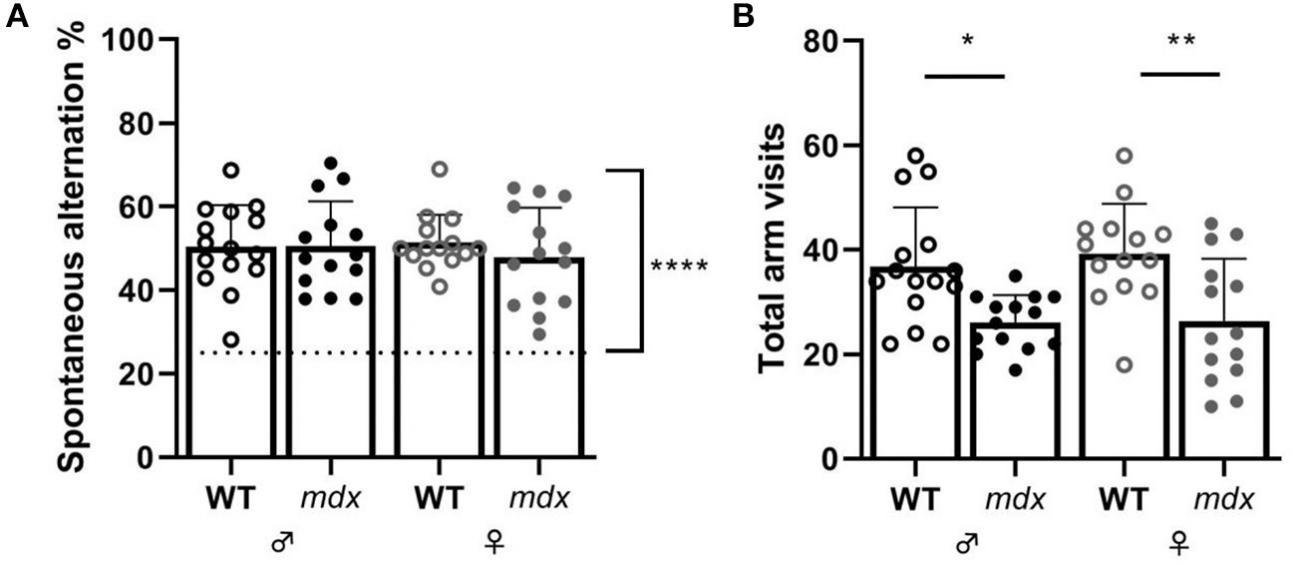
Measures of short-term spatial memory and alternation in the Y-maze. **(A)** Spontaneous alternation percentage. Spontaneous alternation percentage chance level of 25% is represented by a dashed line. **(B)** Total arm visits made during the Y-maze task. Asterisks indicate *P < 0.05, **P < 0.01. WT ♂ *n* = 15*; mdx* ♂ *n* = 14; WT ♀ *n* = 14; *mdx* ♀ *n* = 14.

Spatial working memory was assessed in the Y-maze by measuring the alternation percentage between the three arms of the maze. All mice performed above chance levels during the 10-min trial in the Y-maze (Figure 4A). Wild-type mice visited more arms than *mdx* mice during the test (Figure 4B).

## Discussion

While DMD is primarily characterized by progressive muscle wasting, the cognitive and behavioral implications of the loss of brain dystrophin isoform(s) have been investigated more frequently in recent years. A greater understanding of the pathological consequences of a loss of dystrophin isoforms in the brain of DMD mouse models like the *mdx* mouse, will facilitate the development of therapeutic approaches to treat the DMD brain. In this study, we compared behavioral and cognitive abnormalities between male and female *mdx* mice.

Learning disabilities are common in DMD patients, with incidences ranging from 18.6% to 44% in various cohorts studied (Banihani et al., 2015; Ricotti et al., 2016). Several tests have been designed to study learning abnormalities in mice. In our study, we investigated DL and RL in a PhenoTyper home-cage. We did not find any DL abnormalities in *mdx* mice regardless of their gender. This is in accordance with our previous study in female *mdx* mice (Remmelink et al., 2016). In line with this, other studies have not found abnormalities in DL, assessed in operant procedure tasks in which *mdx* mice received a food-reward for correct actions (Lewon et al., 2017; Dickson and Mittleman, 2019). However, it was found that *mdx* mice actually outperform wild-type mice after a food deprivation period hinting to an additional motivation to perform for a food reward (Lewon et al., 2017). Acquisition learning has also been studied in maze-based assays such as the Morris water maze in which *mdx* mice either did not show any difference in initial learning to find the platform (Sesay et al., 1996; Vaillend and Chaussenot, 2017) or showed an initial reduced performance during the assay but did not show overall impaired learning compared to wild-type mice (Chaussenot et al., 2015). In the Barnes maze (Remmelink et al., 2016) and the Radial maze (Chaussenot et al., 2015) *mdx* and wild-type mice's learning abilities were also comparable.

We previously observed that female *mdx* mice have impaired cognitive flexibility (Remmelink et al., 2016). In that study, four out of the nine female *mdx* mice included did not reach the 80% criterion during the RL phase of the DL/RL test. Furthermore, in this cohort we also found impaired flexibility in the RL phase of the Barnes maze test (Remmelink et al., 2016). In contrast, other studies in which serial reversal learning was either assessed in a food-reward based task and/or in a water maze or radial maze, did not observe impaired flexibility in *mdx* mice (Chaussenot et al., 2015; Dickson and Mittleman, 2019). In our current study, we did not detect a RL deficit in male *mdx* mice, nor could we reproduce our earlier findings of a deficit in the RL task in female *mdx* mice. As the task is fully automated and executed by the mice at night without human interference, we are puzzled about this discrepancy. We here discuss differences between our previous study, our current study and other studies that might have an influence on the discrepancies in behavioral abnormalities seen in the *mdx* mouse model.

Our previous results might have been affected by a small sample size. However, *post-hoc* power calculations showed that the power of that study was larger than 80%, which is typically accepted as a sufficiently powered animal study. Adding to the discrepancy, in the current study we observed a decrease in general activity and subtle differences in sheltering behavior of female mice during the days of spontaneous behavior, which was not detected in the previous study. Meanwhile we have performed the DL/RL tasks in well-powered *n* = 25 *mdx* mice (on a C57BL/6J genetic background), as part of another study, and again did not find RL deficits (manuscript in preparation). Taken together, the fact that two independent studies and our own follow up studies using the DL/RL task did not reproduce these findings show that in the *mdx* mouse reversal learning is unaffected and that our previous observation could have been caused by idiosyncratic factors.

In an attempt to identify these factors, we scrutinized the experimental protocols of the current and previous study. We could not identify obvious differences in the hygiene level of the mice. There were however differences in the breeding scheme and housing conditions. In the current study mice were bred from homozygous wild-type or *mdx* lines while in the previous study we had heterozygous breeding lines that resulted in wild-type, heterozygous and homozygous *mdx* mice. We also noticed that mice were shipped from the breeding location to the test location at a younger age in the previous study (8–11 weeks vs. 8–16 weeks in the present study). Due to the implementation of an additional health screening step in the test facility, mice were housed for an additional 4 weeks in IVC cages, whereas in the previous study mice were housed in open cages upon arrival at the testing facility. There were differences in the number of animals housed per cage. Male mice were housed individually and the number of females per cage was 2–3 mice per cage while in the previous study female mice were housed with 2 mice per cage. The age at which mice were subjected to the DL/RL task was 13 weeks in the previous study, and 18 weeks in the present study. Finally, in the previous study the home-cage testing protocol had an additional 2-day light spot avoidance test in between the 3 days of spontaneous behavior and the DL/RL task, which was not present in the current study. A comparison of the behavioral tests done in the current study and previous study can be found in Supplementary Table 3. In summary, we identified a number of factors that may affect mouse behavior and thereby an *mdx* phenotype.

Besides cognitive flexibility and activity, we also studied anxiety-related behavior in *mdx* mice in the DLB assay. While mice have a natural tendency to explore new spaces, this is hampered in the presence of stressors like bright light (Bourin and Hascoet, 2003). Mice with increased anxiety will therefore show more aversion to brightly lit new spaces than less anxious mice. The DLB is based on this principle and thus a good measure for increased anxiety. In our study, *mdx* mice showed a slight decrease in exploration of the new brightly lit compartment, but this decrease was not significant compared to the wild-types. This is in contrast to our previous study in which we observed an aversion toward the lit area in *mdx* females (Remmelink et al., 2016). This discrepancy cannot be explained by a difference in experimental setup since those are the same. Anxiety has only sporadically been studied in the DLB; one group found that *mdx* males showed increased anxiety-related behavior compared to wild-types (Vaillend and Chaussenot, 2017). However, in one of their previous studies, which used a different DLB setup, they did not see this decrease in time spent in the light compartment (Vaillend et al., 1995). Anxiety has also been studied in the elevated plus-maze in which mice can explore a maze that has two enclosed arms opposite of each other and two open arms. A decrease in exploration of the open arms is indicative for increased anxiety, which was not found in *mdx* males (Sekiguchi et al., 2009; Vaillend and Chaussenot, 2017). Taken together, the underlying cause of discrepancies in findings within and between distinct anxiety tests remains unclear and should be further investigated. Both nature and strength of the anxiogenic stimulus and intrinsic novelty-seeking motivations could be underlying factors.

The Y-maze was used to assess short-term spatial working memory. Rodents have a natural curiosity to explore new areas and thus the spontaneous alternation percentage in the Y-maze gives an indication on the functionality of short-term memory. Mice with a good working memory have a high spontaneous alternation percentage, or a bias toward exploring new arms located to the left or right of them, whereas low spontaneous alternation percentage indicates a poor working memory (Kraeuter et al., 2019). In our study, we observed that all groups performed above chance levels. While the Y-maze requires minimal animal handing due to its continuous nature, it might not be the best experiment to detect (subtle) abnormalities in the spatial working memory of mice (Hughes, 2004; Deacon and Rawlins, 2006). In our previous study, we assessed spatial working memory in the T-maze and observed no differences between *mdx* and wild-type females during a two-day period (Remmelink et al., 2016). Vaillend et al. also performed the T-maze test, using a longer protocol; one sample trial on the first day, two consecutive trials on the second day followed by a final trial either 6 or 24 h later (Vaillend et al., 1995). They observed no abnormalities in the spontaneous alternation percentage at 6 h, however *mdx* mice performed at chance levels while wild-types performed above chance levels at 24 h (Vaillend et al., 1995). Long-term, but not short-term spatial working memory might thus be primarily affected in *mdx* mice.

An outstanding question is which tests are most suitable to study the behavioral abnormalities and to detect efficacy of treatments targeting the brain of *mdx* mice. Ideally, these would be robust and sensitive tests, providing a large therapeutic window and being executed in a similar manner (using identical protocols) within and between labs to allow direct comparisons between outcomes. Generation of Standard operating procedures (SOPs) for a selection of such behavioral tests, as currently available for muscle functionality outcomes on the TREAT-NMD Alliance website (https://treat-nmd.org/research-overview/preclinical-research/experimental-protocols-for-dmd-animal-models/) could facilitate this and improve test reproducibility. Discrepancies described in our current study highlight the need of well-powered studies. This also holds true for tests that are performed in a fully-automated fashion without human interference. Some of the behavioral phenotypes in *mdx* mice appear to be subtle and sensitive to so far unidentified factors that have an impact on mouse behavior prior to the actual testing of the mice. The level of standardization of the present set of studies was obviously not sufficient to reach reproducible results. Given these subtle phenotypes, it is tempting to advocate for standardization of external conditions such as housing, handling etc. This could improve test reproducibility, however, given the uncertainty about which factors to standardize, further standardization might not be successful. Differences in the number of mice housed together can be found over the different studies discussed. Often male *mdx* mice are housed together with 2–5 littermates (*mdx* and wild-types) (Sekiguchi et al., 2009; Chaussenot et al., 2015; Vaillend and Chaussenot, 2017; Dickson and Mittleman, 2019), other times *mdx* and wild-type mice are caged separately but still socially with other male mice (Vaillend et al., 1995). In our study we decided to cage male mice individually. The reasoning behind this comes from observed aggression in male mice and since our mice are individually housed for an extensive period in the PhenoTyper cages, it is deemed more suitable to house male mice individually to reduce the potential aggressive behavior toward cage mates before and after the behavioral testing. *Mdx* mice have been shown to have an increased amount of aggressive encounter with wild-type mice after a period of single housing (Miranda et al., 2015). It is possible that the single housing influenced the mouse behavior and resulted in the observed discrepancy.

Furthermore, if a particular phenotype in mice can only be detected under very specific conditions, the question remains how generalizable any conclusions generated in such a model can be. Here we used *mdx* mice, the most used mouse model for DMD (Yucel et al., 2018). This model is however less severely affected than DMD patients and thus does not fully recapitulate human disease progression. Although muscle pathology is less severe, behavioral abnormalities such as increased stress and freezing response have been detected in this strain (Sekiguchi et al., 2009; Miranda et al., 2015; Comim et al., 2019; Razzoli et al., 2020). Other mouse models for DMD are available and could be interesting to study behavioral and learning abnormalities. The *mdx*^4cv^ mouse model lacks multiple dystrophin isoforms and has a lower number of revertant fibers, fibers containing spontaneously restored dystrophin, than *mdx* mice. Although the muscle pathology appears to be very similar to that of the *mdx* and still less severe than DMD patients, the absence of multiple dystrophin isoforms makes it an interesting model to assess the effect of loss of one or more dystrophin isoforms on cognition and learning abilities (Yucel et al., 2018). Loss of multiple isoforms have been implied to result in a higher incidence of comorbidity with intellectual and emotional disabilities in DMD patients (Banihani et al., 2015; Ricotti et al., 2016). Another interesting mouse model for DMD is the *D2-mdx* mouse model. This mouse model was obtained by crossing *mdx* mice onto a DBA/2J background and resulted in a more severe muscle pathology compared to *mdx* mice (van Putten et al., 2019). Little is known about the effect of loss of full-length dystrophin on the behavioral phenotype of these mice.

To conclude, we do not see differences in spontaneous behavior and cognitive flexibility in *mdx* mice regardless of their gender. Since behavioral differences between *mdx* and wild-type mice seem to be subtle, large sample sizes, the search for more sensitive tests and the availability of standard operating procedures optimized for behavioral testing in *mdx* mice should be encouraged.

## Data Availability Statement

Raw data is publicly available at public.sylics.com.

## Ethics Statement

The animal study was reviewed and approved by Animal Ethics Committee of VU University and performed according to Dutch regulation for animal experimentation, and in accordance with EU Directive 2010/63/EU.

## Author Contributions

MvP, AA-R, and ML contributed to the study conception and design. Experiments were performed by MvP, ML, and BK. Data was analyzed by SE, MvP, ML, and BK. Data was discussed by SE, AA-R, MvP, and ML. The manuscript was written by SE and MvP. All authors contributed to the reviewing and editing of the manuscript.

## Conflict of Interest

ML and BK are full-time employees of Sylics (Synaptologics B.V.), a privately owned company providing services using automated home-cages. AA-R does not disclose anything related to this work. For full transparancy she discloses being employed by LUMC which has patents on exon skipping technology, some of which has been licensed to BioMarin and subsequently sublicensed to Sarepta. As co-inventor of some of these patents AA-R is entitled to a share of royalties. AA-R further discloses being *ad hoc* consultant for PTC Therapeutics, Sarepta Therapeutics, CRISPR Therapeutics, Summit PLC, Alpha Anomeric, BioMarin Pharmaceuticals Inc., Eisai, Astra Zeneca, Santhera, Audentes, Global Guidepoint and GLG consultancy, Grunenthal, Wave and BioClinica, having been a member of the Duchenne Network Steering Committee (BioMarin) and being a member of the scientific advisory boards of ProQR, Eisai, hybridize therapeutics, silence therapeutics, Sarepta therapeutics, and Philae Pharmaceuticals. Remuneration for these activities is paid to LUMC. LUMC also received speaker honoraria from PTC Therapeutics and BioMarin Pharmaceuticals and funding for contract research from Italpharmaco and Alpha Anomeric. Project funding is received from Sarepta Therapeutics. The remaining authors declare that the research was conducted in the absence of any commercial or financial relationships that could be construed as a potential conflict of interest.
