# Supplementary material for: Assessment of Behavioral Characteristics With Procedures of Minimal Human Interference in the mdx Mouse Model for Duchenne Muscular Dystrophy
Source: Front Behav Neurosci. 2021 Jan 20;14:629043. doi: 10.3389/fnbeh.2020.629043 (PMC7855581; doi:10.3389/fnbeh.2020.629043)
Supplement: Supplementary file 1 [file Presentation_1.pdf]

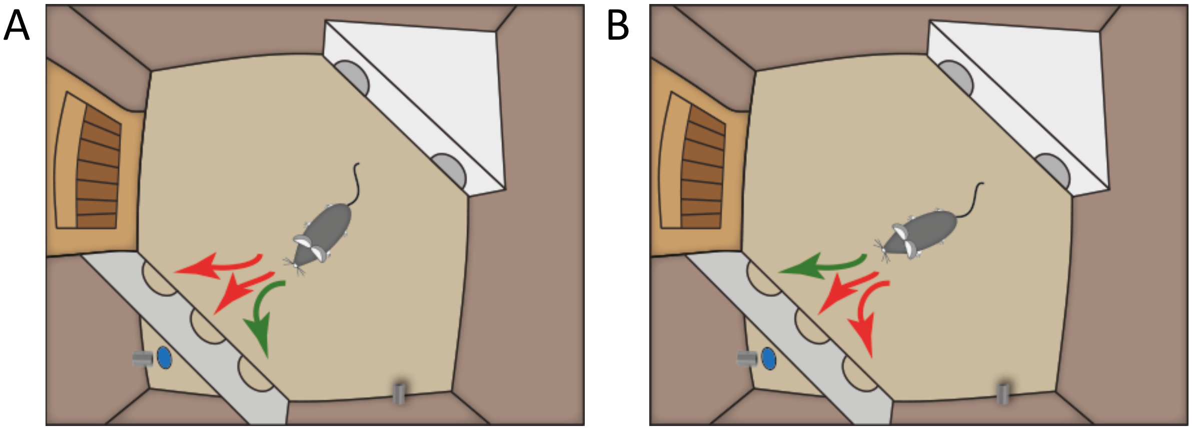

Supplementary figure 1. PhenoTyper home-cage setup during DL/RL task. Pictures from https://www.sylics.com/bioinformatics/testing-protocols/cognitive-tests/reversal-learning/. Used with permission. (A) The PhenoTyper home-cage contains a shelter with two entries in the top right corner, a food grip in the top left, a water bottle in the bottom right and a pellet dispenser in the bottom left corner with the CognitionWall placed before it. During the 2-day discrimination learning phase, mice had to learn that entries through the left entrance was rewarded with a food pellet. Entries through the middle and right entrance were recorded but were not rewarded. (B) During the following 2-day reversal learning phase, entries through the right entrance were rewarded while entries through the left and middle entrance were recorded but left unrewarded.

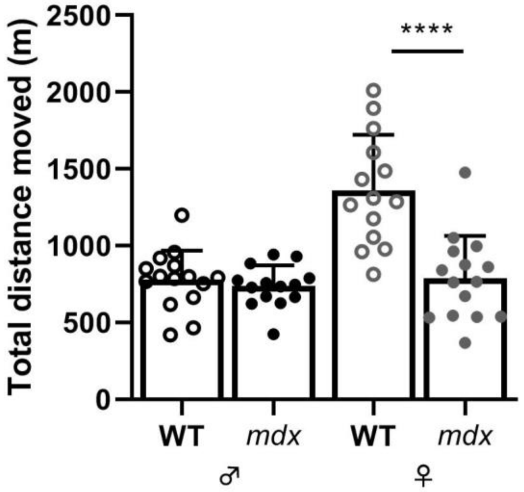

Supplementary figure 2. Total distance moved in meters (m) during the 2.5 day spontaneous behavior assessment. WT; wild-type. **** indicates $P < 0.0001$.

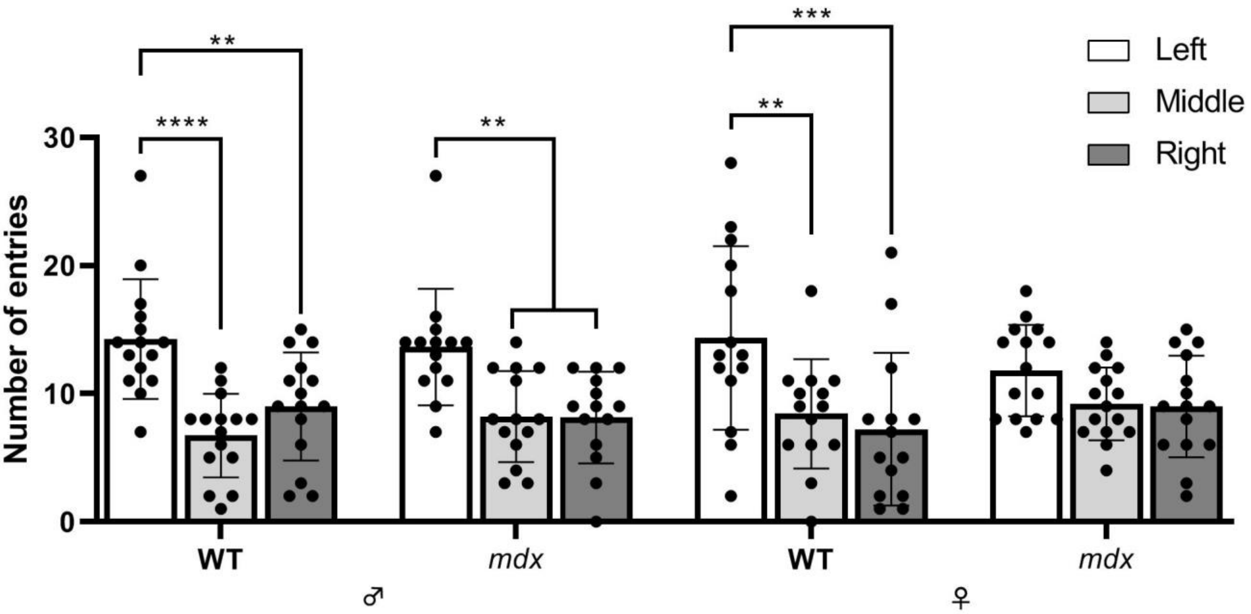

Supplementary figure 3. Side bias for an entrance of the CognitionWall was assessed in the first 30 entries at the start of the DL task. ** $P<0.01$, *** $P < 0.001$, **** indicates $P < 0.0001$.
