# Supplementary material for: Assessment of Behavioral Characteristics With Procedures of Minimal Human Interference in the mdx Mouse Model for Duchenne Muscular Dystrophy
Source: Front Behav Neurosci. 2021 Jan 20;14:629043. doi: 10.3389/fnbeh.2020.629043 (PMC7855581; doi:10.3389/fnbeh.2020.629043)
Supplement: Supplementary file 2 [file Table_1.DOCX]

**Supplementary table 1**. Analysis of total distance moved in meters (m) and key parameters for spontaneous behavior, measured during 2.5 days in the PhenoTyper home-cage environment. The key parameters were defined by Loos et al. (2014). Significance was determined as P<0.05. WT ♂, n= 15; mdx ♂, n=14; WT ♀, n=14, mdx ♀, n=14. #non-parametric analysis

|  | One-way ANOVA | | | Bonferroni’s multiple comparison test | | |
| --- | --- | --- | --- | --- | --- | --- |
|  |  |  |  | ♂ | ♀ |  |
|  |  |  |  |  |  |  |
| **Total distance moved** |  | ***<0.0001*** |  | >0.9999 | ***<0.0001*** |  |
|  |  |  |  |  |  |  |
| **Kinematic parameters of move** |  |  |  |  |  |  |
| Long arrest threshold |  | 0.1222 |  | >0.9999 | >0.9999 |  |
| Long movement max velocity# |  | 0.1237# |  | 0.2419# | >0.9999# |  |
| Long movement threshold# |  | ***0.0003#*** |  | >0.9999# | 0.7625# |  |
| Mean long arrest duration – light# |  | 0.2455# |  | >0.9999# | 0.2779# |  |
|  |  |  |  |  |  |  |
| **Shelter segments** |  |  |  |  |  |  |
| Long shelter visit duration – dark |  | ***0.0331*** |  | >0.9999 | >0.9999 |  |
| Long shelter visit fraction of total visits |  | ***<0.0001*** |  | 0.9785 | 0.3959 |  |
| Long shelter visit threshold |  | ***0.0100*** |  | 0.9682 | ***0.0141*** |  |
| Short shelter visit threshold |  | ***<0.0001*** |  | >0.9999 | ***0.0002*** |  |
|  |  |  |  |  |  |  |
| **Habituation effects across days** |  |  |  |  |  |  |
| Activity duration - habituation ratio dark# |  | 0.5235# |  | >0.9999# | >0.9999# |  |
|  |  |  |  |  |  |  |
| **The effect of light/dark phase** |  |  |  |  |  |  |
| Activity duration - dark/light index |  | 0.1519 |  | 0.7196 | 0.6145 |  |
|  |  |  |  |  |  |  |
| **Anticipation of and response to light** |  |  |  |  |  |  |
| Activity change in anticipation of light # |  | 0.0572# |  | >0.9999# | 0.9889# |  |
| Activity change in response to light |  | ***0.0014*** |  | >0.9999 | 0.9019 |  |
|  |  |  |  |  |  |  |
| **Activity bout characteristics** |  |  |  |  |  |  |
| Activity duration - dark |  | ***0.0087*** |  | >0.9999 | 0.1438 |  |
| Activity duration - light |  | 0.3489 |  | 0.9508 | >0.9999 |  |
| Mean activity duration - dark |  | ***0.0229*** |  | 0.1767 | 0.6202 |  |
| Mean activity duration – light# |  | 0.9101# |  | >0.9999# | >0.9999# |  |
| OnShelter zone number – dark# |  | 0.1002# |  | >0.9999# | 0.9279# |  |
|  |  | #Kruskal-Wallis test |  | #Dunn’s multiple comparison test | |  |

**Supplementary table 2.**  List of all statistical tests performed for each behavioral test. Statistical levels were defined as *P*<0.05.

| **Behavioral test** | **Parameter** | **Groups** | **Statistical test** | ***P*-value** |
| --- | --- | --- | --- | --- |
| Home-cage DL/RL task | Entries to 80% criterion, discrimination learning |  | Mantel-Cox test | 0.9478 |
|  | Entries to 80% criterion,  reversal learning |  | Mantel-Cox test | 0.6982 |
|  |  |  |  |  |
|  | Perseverative errors,  effect genotype |  | Three-way ANOVA | 0.3102 |
|  | Perseverative errors,  effect gender |  | Three-way ANOVA | 0.9393 |
|  | Perseverative errors, effect day |  | Three-way ANOVA | ***<0.0001*** |
|  |  |  |  |  |
|  | Neutral errors, effect genotype |  | Three-way ANOVA | 0.7936 |
|  | Neutral errors, effect gender |  | Three-way ANOVA | 0.1351 |
|  | Neutral errors, effect day |  | Three-way ANOVA | ***0.0097*** |
|  |  |  |  |  |
|  | Total entries | All | One-way ANOVA | 0.1568 |
|  | Total entries | ♂ | Bonferroni’s multiple comparison test | >0.9999 |
|  | Total entries | ♀ | Bonferroni’s multiple comparison test | 0.1673 |
|  | Total distance moved | All | One-way ANOVA | ***0.0133*** |
|  | Total distance moved | ♂ | Bonferroni’s multiple comparison test | >0.9999 |
|  | Total distance moved | ♀ | Bonferroni’s multiple comparison test | ***0.0095*** |
|  |  |  |  |  |
| Dark-light box | Latency to visit the light compartment | All | Kruskal-Wallis test | 0.7274 |
|  | Latency to visit the light compartment | ♂ | Dunn’s multiple comparison test | >0.9999 |
|  | Latency to visit the light compartment | ♀ | Dunn’s multiple comparison test | >0.9999 |
|  | Visits to light compartment | All | Kruskal-Wallis test | 0.0776 |
|  | Visits to light compartment | ♂ | Dunn’s multiple comparison test | 0.5676 |
|  | Visits to light compartment | ♀ | Dunn’s multiple comparison test | 0.5186 |
|  | Time spent in light compartment | All | One-way ANOVA | 0.4179 |
|  | Time spent in light compartment | ♂ | Bonferroni’s multiple comparison test | >0.9999 |
|  | Time spent in light compartment | ♀ | Bonferroni’s multiple comparison test | >0.9999 |
|  |  |  |  |  |
| Y-maze | Spontaneous alternation percentage > 25, WT | ♂ | One sample t-test | ***<0.0001*** |
|  | Spontaneous alternation percentage > 25, WT | ♀ | One sample t-test | ***<0.0001*** |
|  | Spontaneous alternation percentage > 25, *mdx* | ♂ | One sample t-test | ***<0.0001*** |
|  | Spontaneous alternation percentage > 25, *mdx* | ♀ | One sample t-test | ***<0.0001*** |
|  | Total arm visits | All | One-way ANOVA | ***0.0005*** |
|  | Total arm visits | ♂ | Bonferroni’s multiple comparison test | ***0.0322*** |
|  | Total arm visits | ♀ | Bonferroni’s multiple comparison test | ***0.0064*** |

**Supplementary table 3.**  Overview of behavioral test done and the age at which mice underwent the experiments in the current and previous study. *age of mice

|  | Remmelink et al. 2016 | Current study |
| --- | --- | --- |
| Transport | 8-11 weeks* | 8-16 weeks |
| PhenoTyper cages | ~13 weeks | ~18 weeks |
| Spontaneous behavior (2.5 days) |  | ~18 weeks |
| Spontaneous behavior (3 days) | ~13 weeks |  |
| Light-spot anxiety test (2 days) | ~13 weeks |  |
| Discrimination (2 days)/ Reversal learning (2 days) | ~13 weeks | ~18 weeks |
| Avoidance learning task (3 days) | ~13 weeks |  |
| Resting period | ~15 weeks | ~19 weeks |
| Dark-light box (1 day) | ~21 weeks | ~21 weeks |
| Open-field (1 day) | ~21 weeks |  |
| Y-maze (1 day) |  | ~21 weeks |
| T-maze (2 days) | ~21 weeks |  |
| Barnes maze (8 days) | ~22 weeks |  |
